# Neutrophil Homeostasis and Emergency Granulopoiesis: The Example of Systemic Juvenile Idiopathic Arthritis

**DOI:** 10.3389/fimmu.2021.766620

**Published:** 2021-12-13

**Authors:** Bert Malengier-Devlies, Mieke Metzemaekers, Carine Wouters, Paul Proost, Patrick Matthys

**Affiliations:** ^1^ Department of Microbiology, Immunology and Transplantation, Laboratory of Immunobiology, Rega Institute for Medical Research, KU Leuven, Leuven, Belgium; ^2^ Department of Microbiology, Immunology and Transplantation, Laboratory of Molecular Immunology, Rega Institute for Medical Research, KU Leuven, Leuven, Belgium; ^3^ Division of Pediatric Rheumatology, University Hospitals Leuven, Leuven, Belgium; ^4^ European Reference Network for Rare Immunodeficiency, Autoinflammatory and Autoimmune Diseases (RITA) at University Hospital Leuven, Leuven, Belgium

**Keywords:** neutrophil, emergency granulopoiesis, inflammation, systemic juvenile idiopathic arthritis, left shift, cytokines

## Abstract

Neutrophils are key pathogen exterminators of the innate immune system endowed with oxidative and non-oxidative defense mechanisms. More recently, a more complex role for neutrophils as decision shaping cells that instruct other leukocytes to fine-tune innate and adaptive immune responses has come into view. Under homeostatic conditions, neutrophils are short-lived cells that are continuously released from the bone marrow. Their development starts with undifferentiated hematopoietic stem cells that pass through different immature subtypes to eventually become fully equipped, mature neutrophils capable of launching fast and robust immune responses. During severe (systemic) inflammation, there is an increased need for neutrophils. The hematopoietic system rapidly adapts to this increased demand by switching from steady-state blood cell production to emergency granulopoiesis. During emergency granulopoiesis, the *de novo* production of neutrophils by the bone marrow and at extramedullary sites is augmented, while additional mature neutrophils are rapidly released from the marginated pools. Although neutrophils are indispensable for host protection against microorganisms, excessive activation causes tissue damage in neutrophil-rich diseases. Therefore, tight regulation of neutrophil homeostasis is imperative. In this review, we discuss the kinetics of neutrophil ontogenesis in homeostatic conditions and during emergency myelopoiesis and provide an overview of the different molecular players involved in this regulation. We substantiate this review with the example of an autoinflammatory disease, *i.e.* systemic juvenile idiopathic arthritis.

## Introduction

**Figure 1:**
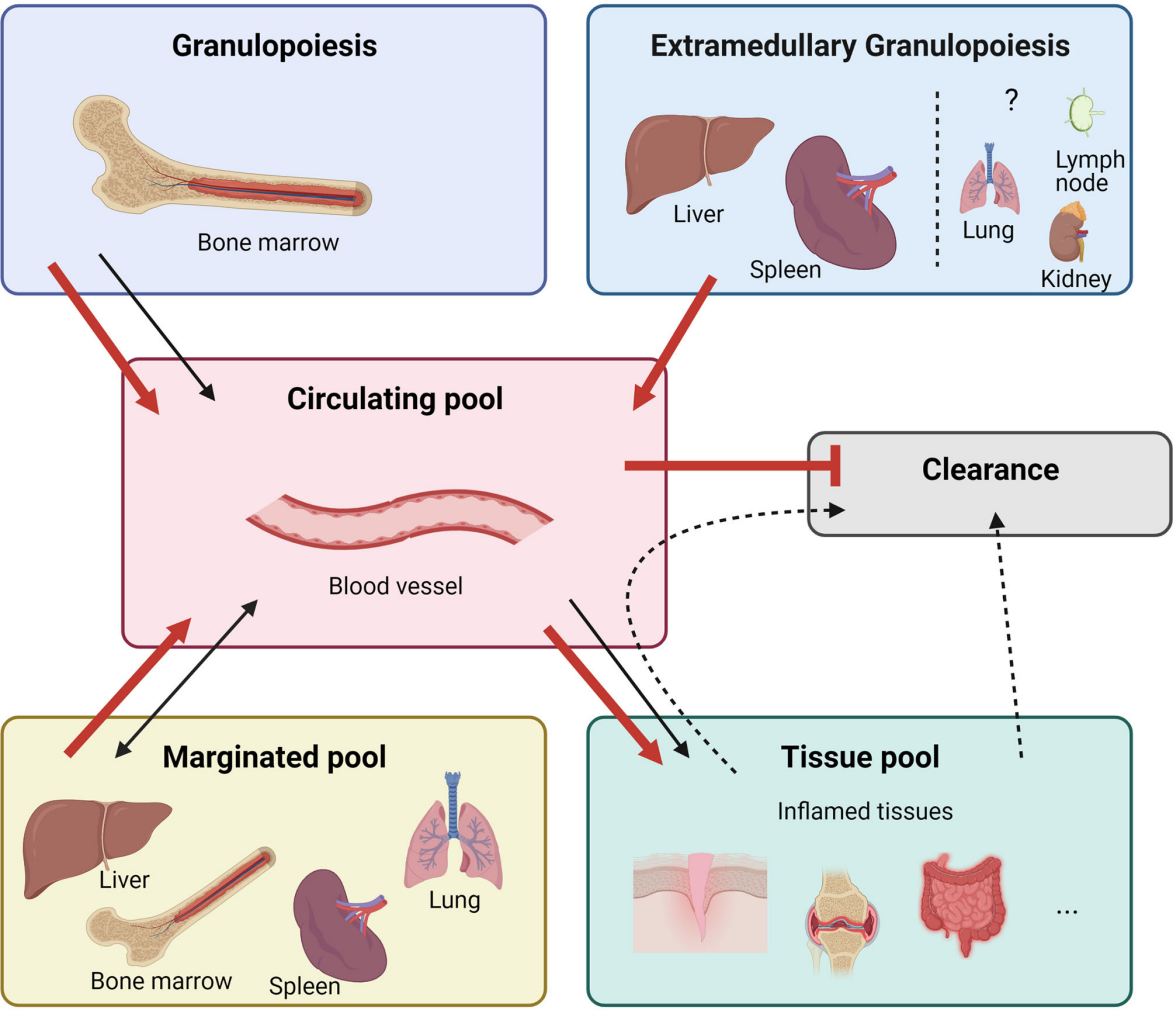
Neutrophil homeostasis is tightly regulated. In homeostatic conditions (black arrows) neutrophils mature in the bone marrow from undifferentiated hematopoietic stem cells (HSCs), replenishing the mitotic stem cell pool that gives rise to mature neutrophils *via* different immature stages of myeloblast, promyelocyte, myelocyte, metamyelocyte, immature band cells, and mature neutrophils. These neutrophils are stored in the bone marrow upon release before entering the blood circulation. Different organs store a pool of neutrophils, referred to as marginated pool, and are forming a reservoir of mature neutrophils. In response to inflammation, neutrophils can exit the bloodstream and enter the tissue pool. Senescent neutrophils are cleared in the bone marrow by stromal macrophages or by tissue-resident macrophages in the periphery. During excessive inflammation (red arrows), neutrophils are massively attracted to the site of inflammation and their clearance is decreased by increasing their lifespan. Demanding the high need for neutrophils, increased numbers of neutrophils are released from the bone marrow. Furthermore, neutrophils are formed and released from extramedullary sites. Cytokine stimulation, including epinephrine, can additionally quickly release neutrophils from the marginated pool.

Neutrophils are the most abundant leukocytes in human blood and protect our bodies from potentially harmful agents (1, 2). The importance of these cells is demonstrated in patients with neutropenia, leukocyte adhesion deficiency, or chronic granulomatous disease who are prone to developing (fatal) microbial infections (3). Traditionally, neutrophils were considered to be homogeneous, simple, and short-lived innate phagocytes mounting rapid - but largely non-specific - antibacterial and antifungal responses. However, discoveries in recent years have revealed the complexity of neutrophil functions including phagocytosis, degranulation, ROS production and NET formation (1, 4, 5). In addition, neutrophils interact with other leukocytes through the production of alarmins (such as S100A8/A9 and S100A12), cytokines and chemokines. Neutrophils have both disease-promoting and disease-limiting properties, indicative of the existence of different neutrophil subsets with unique immunomodulatory functions (6–9). Indeed, the existence of neutrophil subsets with transcriptional, functional, and phenotypic heterogeneity has recently emerged in both humans and mice (10). The production and storage of new neutrophils are tightly regulated. **Figure 1** summarises the different neutrophil regulatory mechanisms that include: the formation of new neutrophils or ‘granulopoiesis’ in the bone marrow (BM), the release of new neutrophils into the circulation, the storage of neutrophils outside the BM (marginated pool), the infiltration of neutrophils into sites of inflammation (tissue pool) and eventually the clearance of (aged) neutrophils (7, 11). Under steady-state conditions, neutrophils are produced in the BM at a rate of 1-2 x 10^11^ cells/day in humans and 1 x 10^7^ cells/day in mice (12, 13). However, during an excessive inflammatory immune response, when there is a high demand for new neutrophils, the life span of neutrophils may increase up to 7 days and the production of new neutrophils may increase tenfold (11, 14). During such excessive neutrophil production - often referred to as ‘emergency granulopoiesis’ - the generation of new cells may additionally take place outside the bone marrow (BM) in a process called ‘extramedullary myelopoiesis’ (15, 16). Diverse autoimmune and autoinflammatory diseases are hallmarked by emergency granulopoiesis. In this review, we provide an overview of the mechanisms and molecules that regulate homeostatic and emergency granulopoiesis. We place special emphasis on neutrophils and cytokines in systemic juvenile idiopathic arthritis (sJIA), which is a rare but severe multifactorial autoinflammatory disease characterised by expansion of neutrophils.

## Regulation of Neutrophil Homeostasis and Major Functions of Neutrophils

### Granulopoiesis: A Process That Takes More Than 10 Days

The BM is the main source of new neutrophils. Neutrophils that mature in the BM can be subdivided into three pools with increasingly restricted proliferation potentials, *i.e.* the stem cell pool, the mitotic pool, and the post-mitotic pool. The hematopoietic stem cells (HSC) of the stem cell pool give rise to the granulocyte-macrophage progenitor (GMP) cells that gradually mature under influence of different cytokines or growth factors into mature neutrophils. Complete neutrophil maturation takes more than 10 days (17, 18).

HSCs localize in dedicated BM niches filled by perivascular cells that express a membrane-bound form of stem cell factor as well as the chemokine CXCL12 (also known as stromal cell-derived factor 1 or SDF-1), which are the ligands for the stem cell antigen CD117 (C-kit) and CXC chemokine receptor 4 (CXCR-4), respectively (19, 20). Two models are proposed that describe the differentiation of these HSCs. In the first classical or hierarchical model, HSCs give rise to committed common myeloid progenitors (CMP) that eventually give rise to GMPs. During the differentiation, their capacity to give rise to other cell types is progressively lost (21–23). In the alternative model, cells rather have a mixed-lineage potential with transcriptional and functional heterogeneity (21). The absence of oligopotent intermediates with mixed cell markers, having a limited differentiation potential, is favouring this second model (24–26). Here, the CMP compartment contains predestined subpopulations that are transcriptionally primed towards becoming either erythrocytes, megakaryocytes, dendritic cells (DCs), monocytes, neutrophils, eosinophils, or basophils (26–28).

**Figure 2:**
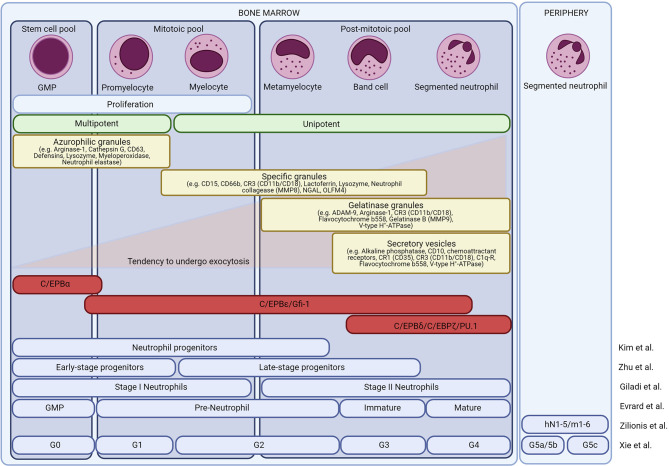
Neutrophil development in the bone marrow. Overview of the different neutrophil subsets in the bone marrow. Morphologically, neutrophils are divided into granulocyte-monocyte progenitor cells (GMPs), promyelocytes, myelocytes, metamyelocytes, band neutrophils, and mature neutrophils. Based on their proliferative and differentiation capacity, the cells are divided into a stem cell pool, a mitotic pool, and a post-mitotic pool. Neutrophil maturation is associated with changes in transcription factors and granule protein expression. At least four types of granules are formed in the neutrophils, each obtaining a unique set of effector molecules. The different granules are released hierarchically, opposite to their formation. Recently, single-cell sequencing or CyTOF reassessed the different neutrophil subsets and different groups have proposed a new neutrophil nomenclature.

The mitotic pool includes promyelocytes and myelocytes. Finally, the most differentiated non-dividing cells include both the immature neutrophils and the mature neutrophils. The different neutrophil precursors can be distinguished microscopically. Promyelocytes are defined as large cells with an oval nucleus and dark cytoplasm. Myelocytes have a less dense cytoplasm and a round-shaped nucleus. Metamyelocytes, band cells, and mature neutrophils are relatively small cells and have a clear cytoplasm. In humans, metamyelocytes and band cells have a kidney-like nucleus whereas in mice these cells have a doughnut-like, band-shaped nucleus. Finally, mature neutrophils are characterised by their segmented nuclei (29–34). The different neutrophil precursors are shown in **Figure 2**.

**Figure 3:**
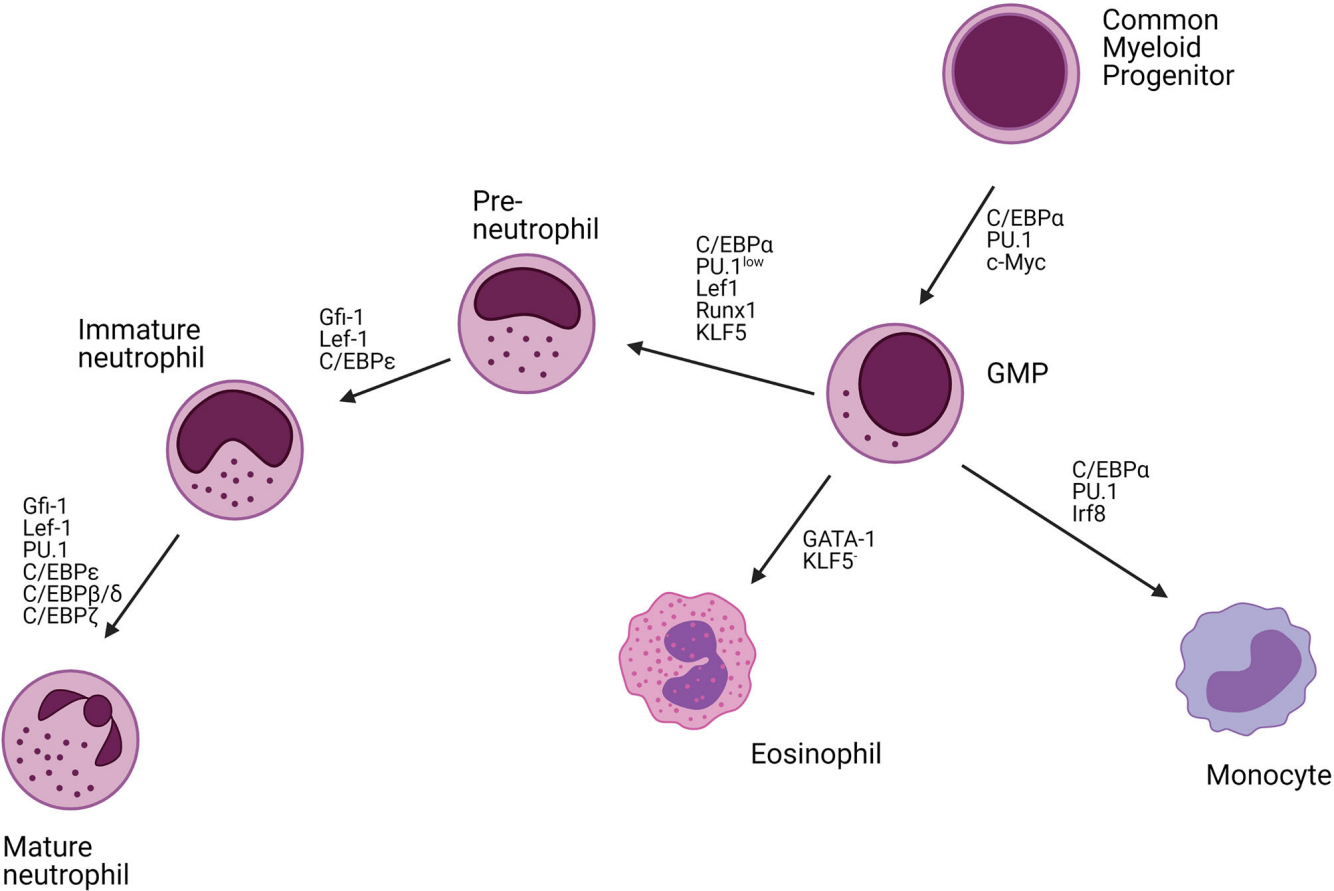
Transcription factors regulating neutrophil differentiation. Neutrophils mature from hematopoietic stem cells (HSCs) in the bone marrow (BM). These HSCs are self-reviewal and can differentiate into all immune cells. The common myeloid progenitor (CMP) cells give rise to the myeloid lineage including neutrophils, monocytes, and eosinophils. C/EBPα is the main transcription factor driving myeloid differentiation. Together with PU.1, and c-Myc, C/EBPα drives granulocyte-monocyte progenitor cell (GMP) differentiation. High levels of C/EBPα, PU.1, and Irf8 further drive monocyte development, high levels of GATA-1 eosinophil development, whereas C/EBPα, Lef1, Runx1, KLF5 together with low expression of PU.1 enhance neutrophils development. Neutrophil maturation is further driven by Gfi-1, Lef-1, C/EBP transcription factors (C/EBPϵ, C/EBPβ, C/EBPδ, C/EBPζ), and PU.1. C/EBP, CCAAT/enhancer-binding protein; Irf8, Interferon regulatory factor 8; Lef1, Lymphoid enhancer-binding factor 1; Runx1, Runt-related transcription factor 1; KLF5, Krüppel Like Factor 5; GFI1, Growth Factor Independent 1 Transcriptional Repressor.

The differentiation of neutrophils requires dynamic changes in the activity of specific transcription factors. The main transcription factors driving granulopoiesis are CCAAT/enhancer-binding protein (C/EBP) α and PU.1 (**Figure 3**). High expression of C/EBPα or PU.1 is associated with commitment to the granulocyte or monocyte lineage, respectively. Runx1 and lymphoid enhancer-binding factor 1 (Lef1) are responsible for regulating the expression of C/EBPα with defects in their activities resulting in a neutrophil maturational block (33, 35, 36). C/EBPα itself negatively regulates the expression of cMyc. Consistently, C/EBPα mutant mice have an early block in granulocyte differentiation (37–39). Another major transcription factor involved in neutrophil development is Krüppel-like factor 5 (KLF5), which is mainly active during early developmental stages and controls neutrophil production at the expanse of eosinophils (40). In addition, growth factor independent-1 (Gfi-1) also drives the initial neutrophil development and represses the monocyte-promoting transcription factors PU.1 and Irf8 (26, 41–47). Mutations in Gfi-1 block neutrophil maturation at the promyelocyte stage (33, 36, 46). The expression of Gfi-1 and C/EBPα decreases from the myeloblast stage and is associated with an increased expression of C/EBPϵ that peaks at the myelocyte stage. C/EBPϵ regulates the transition from promyelocytes to myelocytes and represses genes involved in cell cycling. The deletion of C/EBPϵ leads to neutrophil progenitor arrest and neutropenia (26, 48–51). Further myelocyte maturation is driven by an upregulation of C/EBPβ, C/EBPγ, C/EBPδ, and C/EBPξ.

The different maturational stages are accompanied by the sequential formation of primary (azurophilic), secondary (specific), tertiary (gelatinase), and secretory granules (34). Primary granules are mainly formed during the myeloblast stage (GMP, promyelocyte) and the main granule proteins include myeloperoxidase (MPO), α-defensins, bactericidal/permeability-increasing protein (BPI), and distinct serine proteinases such as elastase or cathepsin G (**Figure 2**). The main secondary granule proteins are lactoferrin (LTF) and lysozyme and are mainly formed during the myelocyte stages (myelocyte, metamyelocyte). Tertiary granules are rich in gelatinases and are formed during the band cell stage (banded immature neutrophils). Secretory vesicles are mainly generated during the final stages of neutrophil maturation and contain early activation-related proteins that facilitate neutrophil adhesion and migration. Upon neutrophil activation, the different granules are hierarchically released according to the “formed-first-released-last model” (52).

Novel insights on granulopoiesis were recently provided by innovative approaches such as single-cell RNA sequencing (scRNAseq) and mass cytometry (CyTOF) and have suggested that the classical nomenclature may require adjustment to accurately represent the true heterogeneity of neutrophils. Evrard et al. performed CyTOF analysis on murine BM cells and revealed the existence of three neutrophil subsets including a proliferative neutrophil progenitor group (preNeu), and non-proliferating immature and mature groups (49). PreNeu express CD117 (c-kit) but do not express markers for other leukocyte lineages. Phenotypically, preNeu were defined as Lin^-^c-kit^int^CD11b^+^CXCR4^+^ cells containing primary and secondary granules. The human counterparts of the preNeu subset were defined as being Lin^-^CD34^-^CD101^-^CD15^+^CD66b^+^ cells. Immature and mature neutrophils can be discriminated based on the distinct expression profiles of CXCR2 and CD101. Immature neutrophils are Lin^-^c-kit^-^CD11b^+^CXCR4^-^CXCR2^-^CD101^-^ (or Lin^-^CD11b^+^CD115^-^Ly6G^low^Ly6B^int^) and are mainly expressing secondary granule proteins. Mature neutrophils are CD11b^+^CD115^-^Ly6G^+^CXCR2^+^CD101^+^ and express gelatinase granule proteins (49). In humans, immature and mature neutrophils are defined as Lin^-^CD34^-^CD101^+^CD15^+^CD66b^+^CD10^-^CD16^-^ and CD101^+^CD15^+^CD66b^+^CD10^+^CD16^+^, respectively (53, 54). Kim et al. confirmed the existence of a murine neutrophil precursor and defined these cells as Lin^-^c-kit^+^CD11b^+^Ly6G^low^Ly6B^int^CD115^-^GFI1^+^ cells (55). Later Zhu et al. showed that two populations of neutrophil precursor cells can be distinguished based on the expression of CD34. Early-stage and late-stage precursor cells were defined as c-kit^+^Gfi1^low^CEBPA^hi^Ly6G^low^ and c-kit+Gfi1^high^CEBPA^low^Ly6G^+^ cells, respectively (56).

### Circulating Pool of Neutrophils in Blood

**Figure 4:**
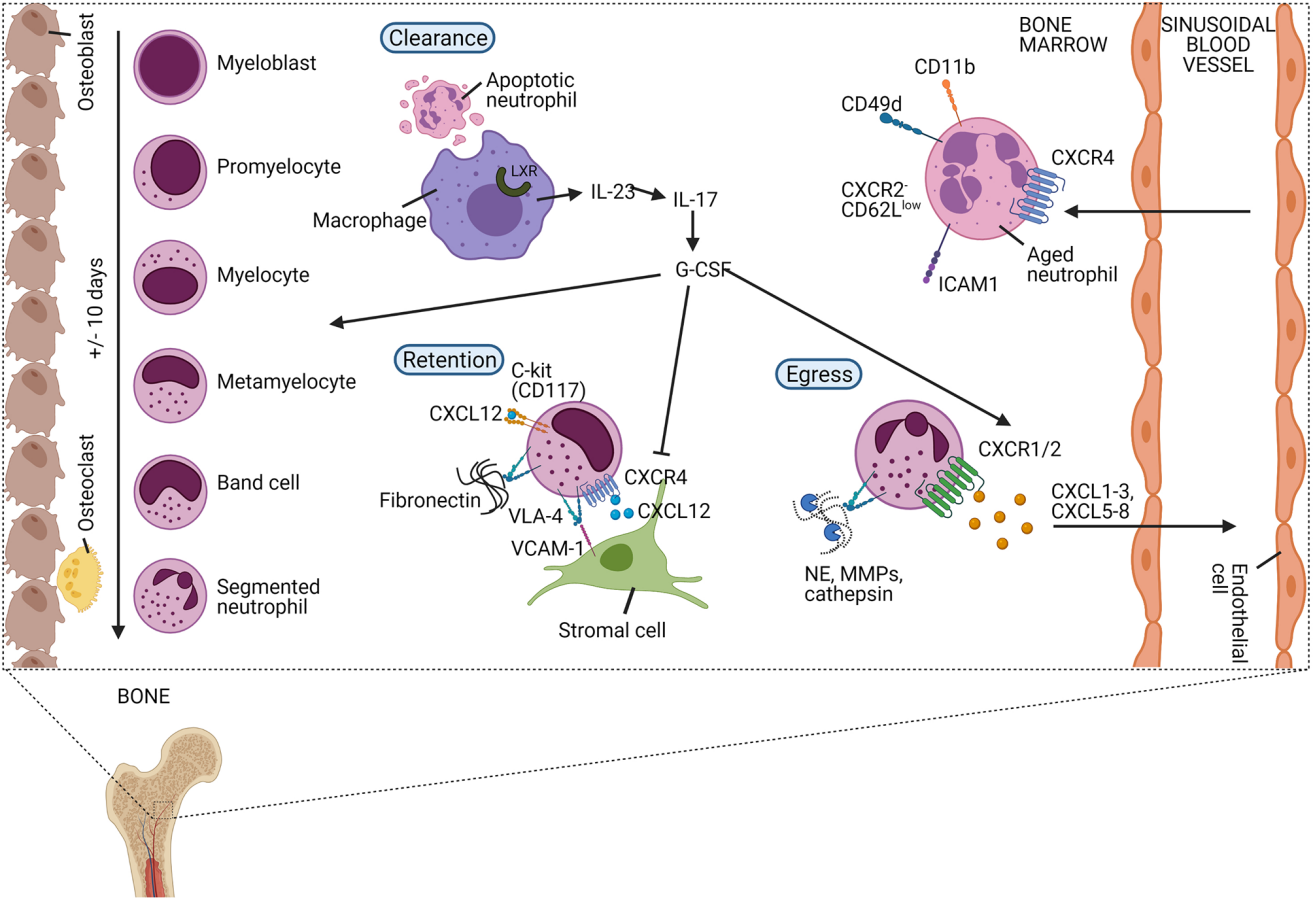
The release of neutrophils from the bone marrow is tightly regulated in homeostatic conditions. In the bone marrow, neutrophils differentiate from undifferentiated hematopoietic stem cells (HSCs) into mature neutrophils *via* different immature stages of myeloblast, promyelocyte, myelocyte, metamyelocyte, immature band cells, and mature segmented neutrophils. Neutrophils are retained in the bone marrow (BM) *via* the homing CXCR4 receptor or the stem cell antigen CD117 (C-kit) which both bind CXCL12, released by stromal cells. Additionally, the integrin very late antigen 4 (VLA-4) expressed on neutrophils are retaining neutrophils in the BM by binding the adhesion molecule vascular cell adhesion protein-1 (VCAM-1) or fibronectin. Granulocyte colony-stimulating factor (G-CSF) is an important growth factor that regulates the proliferation, differentiation, and egress of neutrophils in the BM. It aborts the CXCR4/CXCL12 interactions and releases neutrophilic chemoattractants including CXCL1-3 and CXCL5-8 that bind CXCR2. During the release of neutrophils from the BM, granule proteins are released degrading the fibronectin and mediating trans-endothelial transport. Aged neutrophils are characterised by the expression of diverse receptors including upregulated CXCR4, ICAM1, CD11b, CD49d, and low expression of CXCR2 and CD62L. Aged neutrophils migrate back to the BM where they are engulfed by macrophages. Upregulation of the liver X receptor (LXR) family induces the expression of interleukin-23 (IL-23) that may enhance the release of new neutrophils, balancing the homeostasis of neutrophils, *via* the IL-23/IL-17/G-CSF-axis.

Mature neutrophils can be stored in the BM and are liberated into the circulation upon appropriate stimulation (**Figure 4**) (1, 7). Recent scRNA-seq experiments have exposed an additional layer of complexity by showing that neutrophils can enter the peripheral blood without going through the most mature stage first, entering the circulation as immature cells (57). The balanced action of the chemokine receptors CXCR4 and CXCR2 tightly regulates the release of neutrophils. Upregulation or downregulation of CXCR2 and CXCR4, respectively, is associated with egress from the BM (58). CXCR4 is the main receptor for CXCL12, which is expressed by the stromal cells of the BM and retains developing neutrophils within the BM (11). Enhanced activity of CXCR4 delays the release of mature neutrophils from the BM as seen in patients with WHIM (‘warts, hypogammaglobulinemia, infections, and myelokathexis’) syndrome, a rare primary immunodeficiency disorder (59). Also, the adhesion molecule vascular cell adhesion molecule (VCAM)-1, expressed by the BM epithelial cells, promotes retention of neutrophils within the BM *via* its interaction with the integrin very late antigen (VLA)-4 on neutrophils (11). In contrast, human CXCR2 - which recognises CXCL1, CXCL2, CXCL3, CXCL5, CXCL6, CXCL7, and CXCL8 - stimulates the release of neutrophils from the BM. Granulocyte colony-stimulating factor (G-CSF) further promotes neutrophil mobilization by lowering CXCL12 production (60) and CXCR4 expression (61) and by increasing the amounts of mobilising signals (*e.g.* CXCL1) (62). The release of neutrophils outside the BM is accompanied by the release of granule proteins including MMP-9, paving the transendothelial/transcellular way through the sinusoidal endothelium (63, 64). Moreover, in human, the most potent chemokine activator of CXCR2, i.e. CXCL8 is further potentiated by N-terminal truncation by MMP-9 creating a positive feedback loop and inhibition of CXCL8 with neutralizing anti-MMP-9 antibodies inhibited CXCL8-induced mobilization of progenitor cells from bone marrow in monkeys (65, 66). Interestingly, the transit from the BM to the peripheral blood is associated with large transcriptomic and epigenetic differences (67).

### Marginated Pools of Neutrophils in Organs

Margination refers to the prolonged transit of neutrophils through organs, which results in discrete intravascular (marginated) pools. These can be found within the spleen, liver, and BM. Lung margination may be specific only for certain species such as primates, mice, and dogs (1, 58, 68–70). During infection or inflammation, cytokine or epinephrine (adrenaline) stimulation can quickly release the marginated neutrophils into the circulation (71–73).

### Extravasation of Neutrophils Into Inflamed Tissues

**Figure 5:**
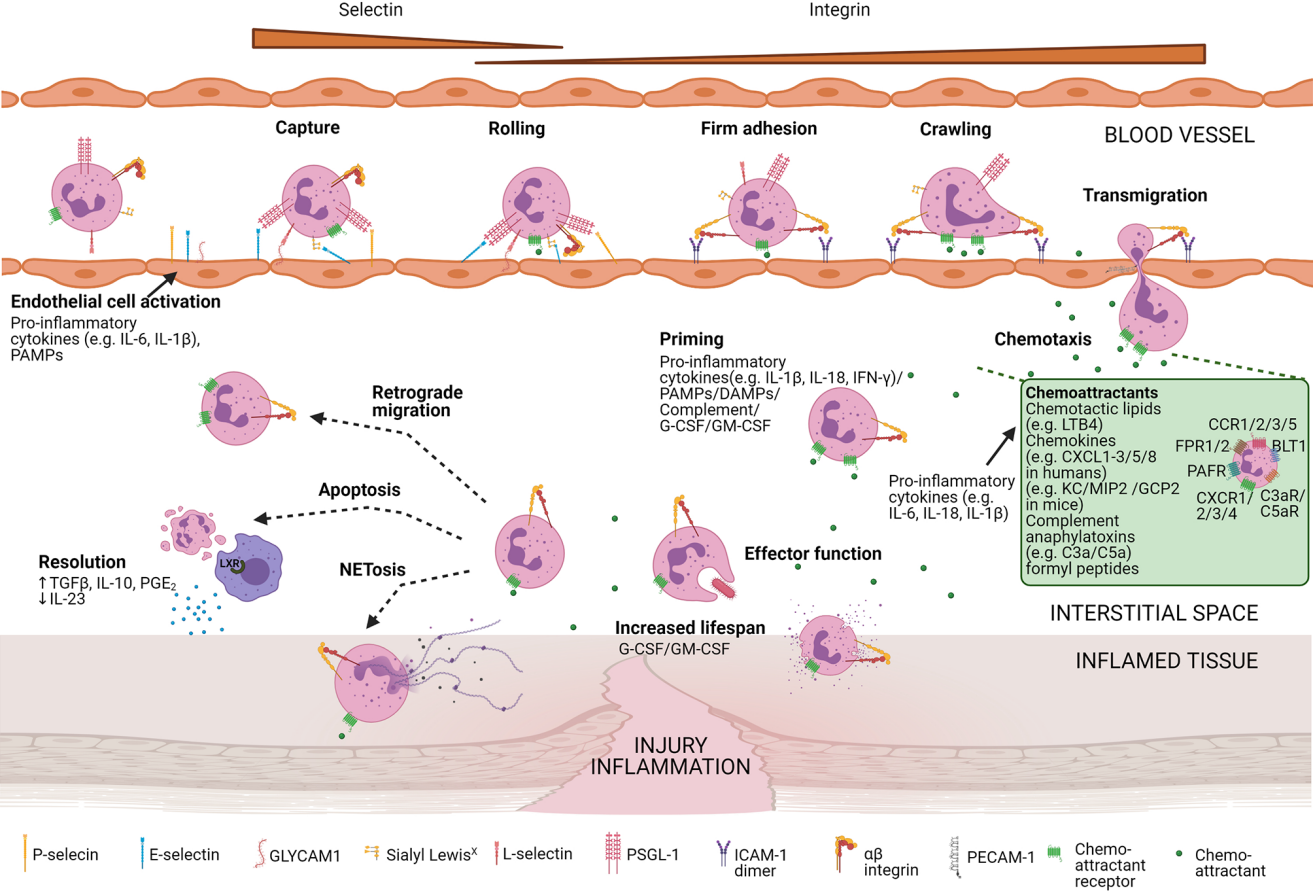
Neutrophil recruitment towards the site of inflammation. Neutrophil mobilisation and internalisation involve different steps including the capture of the cells, rolling, firm adhesion, crawling, and transmigration. Diverse neutrophil surface receptors are involved in this process. Pro-inflammatory cytokines or pathogen-associated molecular patterns (PAMPS) activate endothelial cells, allowing them to express adhesion molecules (e.g. P-selectins and E-selectins). These adhesion molecules bind mucins expressed on the neutrophil surface capturing and slowing the neutrophil. Slow rolling of the cells allows chemoattractants to bind their respective chemoattractant receptors. Subsequent signalling results in integrin activation and eventually firm adhesion. Next, neutrophils are crawling along with the endothelial cells and transmigrate at cell-cell junctions (paracellular migration) or by endothelial cell bodies (transcellular migration). Once migrated, neutrophils are further attracted towards the centre of inflammation by a gradient of chemoattractants. An overview of the main chemoattractants is shown in the green box. Chemoattractants are mainly released by pro-inflammatory cytokines. At the focus of inflammation, neutrophils are primed by proinflammatory cytokines, PAMPs, damage-associated molecular patterns (DAMPs), complement molecules, and/or growth factors enhancing their effector function and additionally increases their lifespan. *In vivo* studies in animals suggest that extravasated neutrophils can back-migrate (retrograde migration). Otherwise, neutrophils undergo NETosis or apoptosis. Apoptotic neutrophils are engulfed by tissue-resident macrophages that upregulate transcription factors of the liver X receptor (LXR) family, potentiating resolution at the site of inflammation.

Effective pathogen elimination requires the presence and activation of neutrophils at the right location in the body. Neutrophils need to enter the inflamed tissue *via* extravasation, a process that is coordinated by selectins, integrins, and soluble mediators including proteases and chemoattractants (**Figure 5**). The different chemoattractants are classified into chemotactic lipids [*e.g.* leukotriene B4 (LTB4)], chemokines [CXCL1 to CXCL3 and CXCL5 to CXCL8 in humans and KC, macrophage inflammatory protein-2 (MIP2), and granulocyte chemotactic protein-2 (GCP-2) in mice], complement anaphylatoxins (C3a and C5a), and formyl peptides [*e.g* N-formylmethionyl-leucyl-phenylalanine (fMLF)]. Their specific roles in the regulation of neutrophil migration and activation were recently reviewed by Metzemaekers et al. (74–76).

Neutrophil extravasation encompasses four well-defined steps. First, pro-inflammatory cytokines (including tumour necrosis factor alpha (TNF-α), interleukin (IL)-1β, and IL-17) or stimulants of bacterial origin (including LPS) induce the upregulation of P-selectins and E-selectins on endothelial cells. These adhesion molecules can bind to glycoproteins *e.g.* P-selectin glycoprotein ligand-1 (PSGL-1) present on the surface of neutrophils, mediating rolling of the latter along the endothelium. Next, chemoattractant-induced signalling and interaction between L-selectin (CD62L) and its ligands (*e.g.* GlyCAM-1) on endothelial cells can activate integrins such as lymphocyte function-associated antigen 1 (LFA-1) on neutrophils. LFA-1 binds to ICAM1 and ICAM-2 on endothelial cells and mediates firm neutrophil adhesion and arrest. Afterwards, neutrophils crawl along the endothelium and exit the blood vessel preferentially *via* the paracellular route. Neutrophils may also follow a transcellular path directly through the endothelial cell body without the loss of the integrity of the plasma membrane of either cell. Finally, neutrophils further migrate towards increasing concentrations of chemoattractants (1, 77–83).

### Neutrophilic Functions at the Site of Inflammation

**Figure 6:**
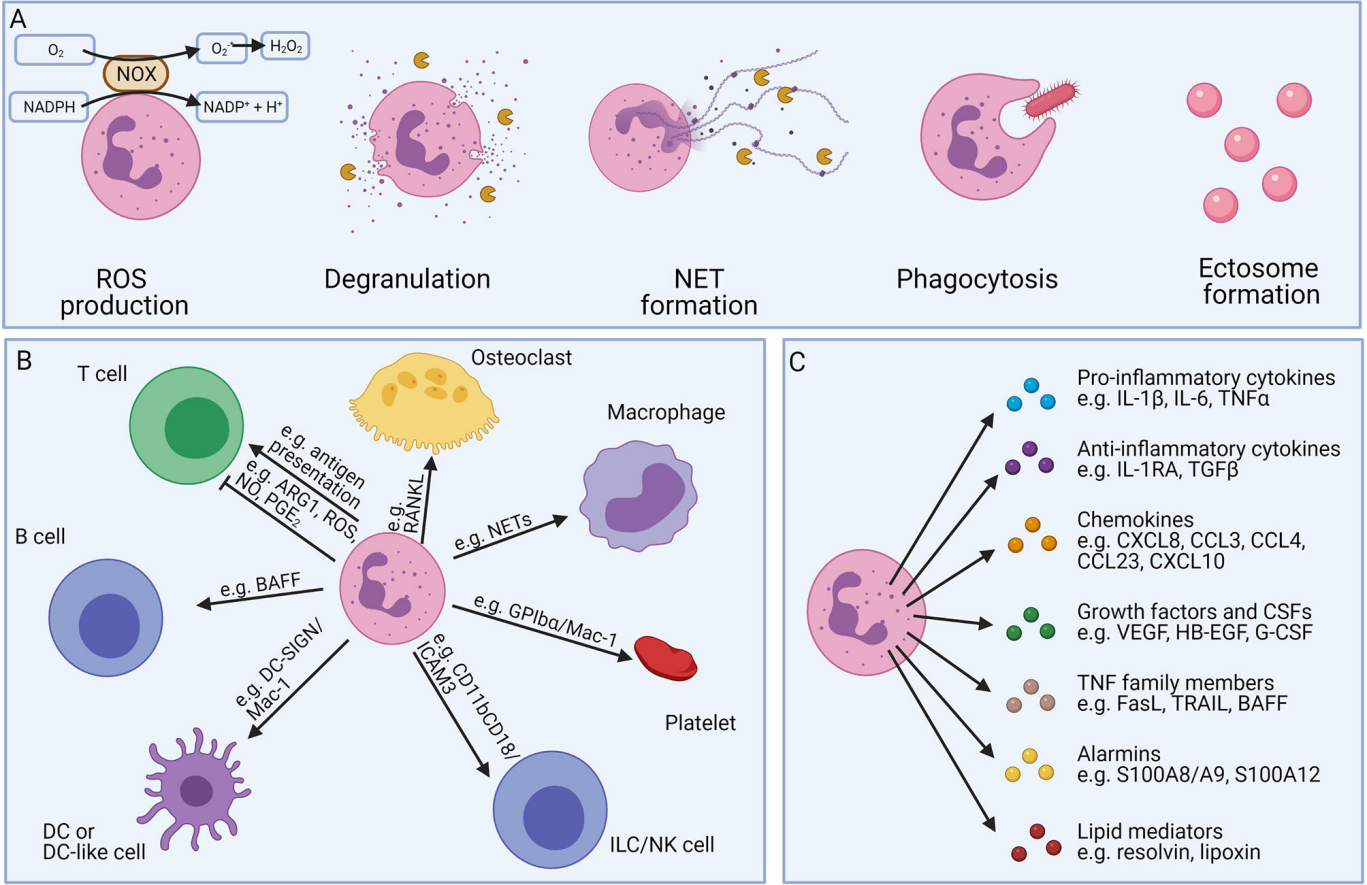
Overview of the different neutrophil effector functions. Neutrophils possess different defence mechanisms including both oxidative and non-oxidative mechanisms **(A)**. Neutrophils can produce reactive oxygen species (ROS) and release soluble mediators stored in pre-made granules (degranulation). Neutrophils show a unique form of cell death, called NETosis, where neutrophils expel neutrophil extracellular traps (NETs), consisting of decondensed DNA together with histones, cytokines, and granule proteins. Furthermore, neutrophils are professional phagocytes able to engulf foreign particles for internal digestion. Neutrophils can also transform into anuclear cytoplasts (ectosomes), regulating the inflammatory microenvironment, intercellular communication, and exerting antimicrobial functions. Neutrophils interact with different cells of both the innate and adaptive immune systems **(B)**. Neutrophils can carry antigens and act as antigen-presenting cells, activating T cells. In contrast, myeloid-derived-suppressor neutrophils can inhibit the proliferation of T cells. Neutrophils can stimulate the maturation of antibody-producing B cells and play an essential role in the generation of natural killer (NK) cells in the bone marrow. Furthermore, neutrophils may also interact with platelets, macrophages, dendritic cells (DCs), or osteoclasts. Neutrophils also release a bunch of soluble mediators **(C)**. Although the release per individual cell is limited, neutrophils are an important source, due to their high abundance in the blood.

Neutrophils have diverse functions which are illustrated in **Figure 6** (1, 4, 5). A full description of the different neutrophilic functions falls out of the scope of this review and has been provided elsewhere (1). Main antimicrobial functions include: ROS production, degranulation, neutrophil extracellular trap (NET) formation, phagocytosis and ectosome formation (**Figure 6A**). Whereas the degranulated granule proteins may tag (*i.e.* by the binding of cationic antimicrobial peptides such as defensins or cathelicidins) or eliminate extracellular pathogens [*i.e.* by neutrophil elastase (NE), MPO, or lactoferrin], they may also induce tissue damage and neutrophil migration (*i.e.* by MMP-8 or MMP-9) (1, 47, 78, 84, 85). The secreted granule proteins also influence the inflammatory activities of the neighbouring cells and promote cytokine, chemokine, ROS production, cell activation, or cell extravasation.

Next to the important antimicrobial properties, neutrophils can also directly or indirectly interact with other immune cells (including T cells, B cells, NK cells, macrophages, and DCs) and are critical in establishing a good immune response (summarised in **Figure 6B**). Neutrophils can secrete both pro-inflammatory cytokines (e.g. TNF-α, IL-18, and IL-1β), anti-inflammatory cytokines (i.e. IL-10, and IL-1RA), and chemokines (e.g. CCL2, CXCL8, CXCL9, CXCL10, and CCL20), further recruiting new immune cells to the site of inflammation (86–91). Despite the relatively low amounts of cytokines/chemokines produced by neutrophils and their limited capacity for *de novo* protein synthesis, neutrophils and their activation products can play significant roles during inflammation since they are usually overwhelming in terms of absolute cell numbers (92). The neutrophil-derived proteases may additionally modulate the signaling network *via* their cytokine and chemokine processing capacity (93–95). Neutrophils also produce growth factors, alarmins, and angiogenic factors (G-CSF, S100 proteins, and VEGF) *via* which they may influence different biological processes (86, 96, 97) or may induce immunoglobulin class switching and somatic hypermutations in B cells by the secretion of B-cell activating factor (BAFF), a proliferation-inducing ligand (APRIL), also known as TNF ligand superfamily member 13 (TNFSF13), and IL-21 (98–101). Neutrophils can also regulate the inflammatory outcome by changing the composition of the secreted lipids (**Figure 6C**) (4).

At the site of inflammation, neutrophils can be primed by cytokines (IL-1β, IL-6 or TNF-α), pathogen-associated molecular patterns (PAMPs; *e.g.* LPS), damage-associated molecular patterns (DAMPs; *e.g.* ATP), or complement-opsonized particles *via* their vast repertoire of cytokine receptors and pathogen recognition receptors (PRRs) (102). Primed neutrophils are phenotypically defined by increased CD16 or CD11b/CD18 expression levels and reduced expression of CD62L. In comparison with quiescent cells, primed neutrophils display a more aggressive action upon subsequent activation with a second inflammatory stimulus, illustrated by enhanced ROS production, degranulation, phagocytosis, and an increased tendency to release NETs (103).

### Apoptosis and Neutrophil Clearance

Under homeostatic conditions, senescent ‘aged’ neutrophils upregulate CXCR4 and CD11b while downregulating CD62L (104, 105). Aged neutrophils transmigrate into the BM where they are cleared by the resident stromal macrophages. This results in the induction of transcription factors belonging to the liver X receptor (LXR) family. Macrophages and LXRs are essential components for the modulation of the hematopoietic niche and may cause G-CSF production and downregulation of CXCL12, which subsequently result in an increased neutrophil mobilisation (106). Using similar mechanisms, hypocellularity (*e.g.* during antibody depletion), can also be sensed in the BM. In addition, circadian rhythm and food intake *via* microbiota-derived signals also regulate the number, maturation, and gene expression program of neutrophils. A full description of the intrinsic clockworks or diurnal rhythm variations in systemic and local factors falls out of the scope of this review [reviewed in (107–110)].

After fulfilling their effector functions at the site of inflammation, neutrophils may undergo suicidal NET formation, necrosis, or apoptosis. Apoptotic neutrophils can be phagocytosed by resident macrophages and subsequently upregulate LXR (111, 112). This induces a reduced IL-23 production and subsequent reduced IL-17 and G-CSF that terminates the inflammatory recruitment of additional neutrophils, tempering inflammation and restoring homeostasis. Furthermore, it stimulates the secretion of the anti-inflammatory cytokines TGF-β and IL-10, which further decrease the neutrophil chemo-attraction and activation (6).

## Emergency Granulopoiesis in sJIA

### Emergency Granulopoiesis and Extramedullary Myelopoiesis

During excessive inflammation, neutrophil homeostasis is disturbed. Neutrophils are massively attracted to the site of inflammation, their clearance is decreased, and their lifespan is increased. Cytokine stimulation can quickly release neutrophils from the marginated pool and the high demand for new neutrophils results in a massive generation of new neutrophils (outside the BM) described as emergency granulopoiesis and extramedullary myelopoiesis respectively (**Figure 1**, red arrows).

**Figure 7:**
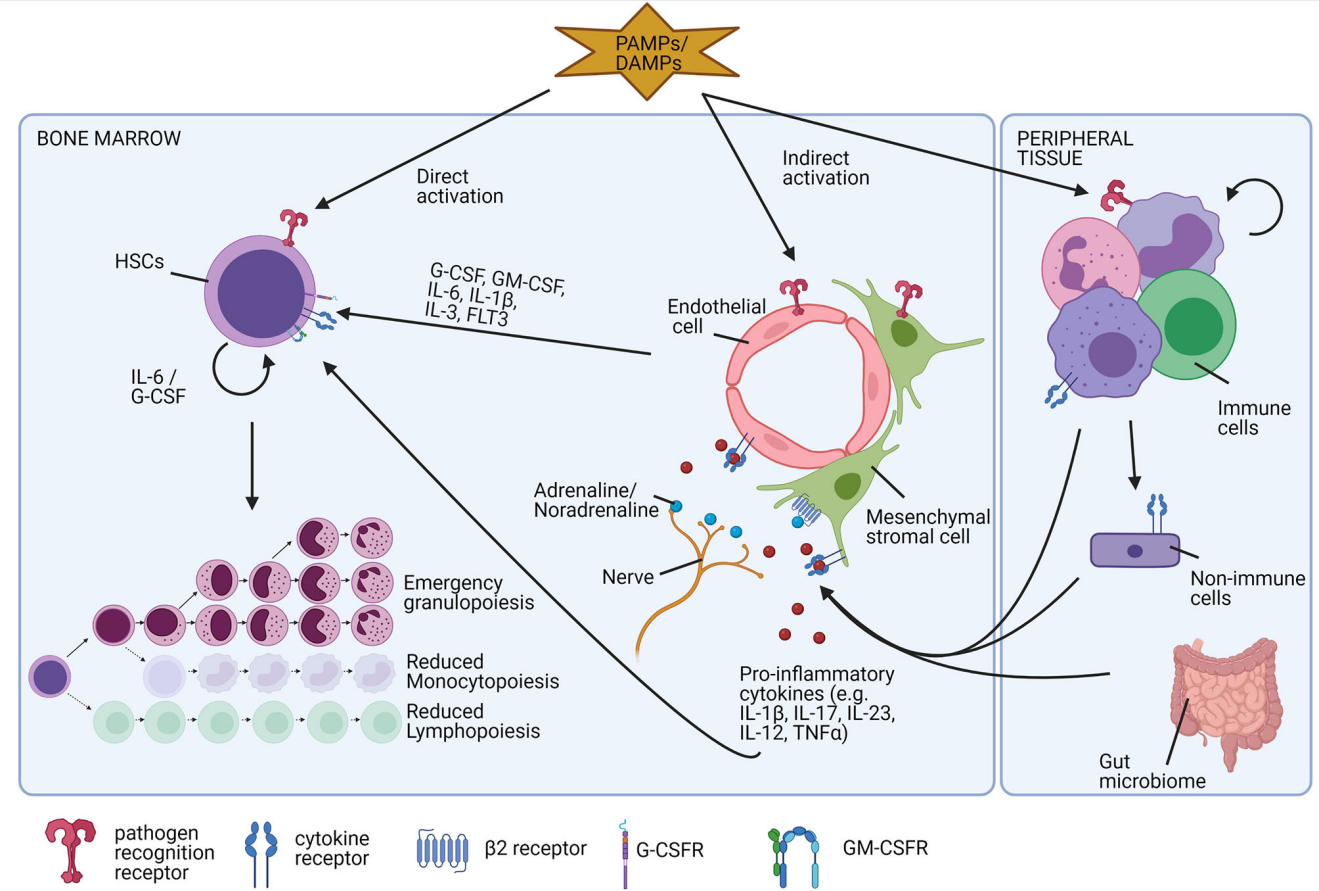
Factors involved in the induction of emergency granulopoiesis. During excessive inflammation, there is a high need for new neutrophils (emergency granulopoiesis). Different direct and indirect mechanisms are involved in the differentiation and release of new neutrophils. Damage-associated molecular patterns (DAMPs) or pathogen-associated molecular patterns (PAMPs) bind diverse pathogen recognition receptors, expressed on diverse cell types. In hematopoietic stem cells (HSCs), binding directly stimulates the release of interleukin-6 (IL-6) and granulocyte colony-stimulating factor (G-CSF), inducing their proliferation and differentiation into neutrophils. Indirectly, the molecular pattern molecules bind endothelial cells or stromal cells, providing an important source of pro-inflammatory molecules and growth factors. Additionally, these endothelial cells or mesenchymal cells are stimulated by pro-inflammatory cytokines released by immune cells or non-immune cells of the periphery that encounter invading pathogens or cell damage. The neuronal release of adrenalin or noradrenalin additionally stimulates the release of new neutrophils. Finally, also food intake and the gut microbiome tightly regulate neutrophil homeostasis.

Emergency granulopoiesis involves a series of conserved cascading events and is especially well documented during infection (113, 114). The different steps involved are summarised in **Figure 7**. Emergency granulopoiesis is characterised by an increased release of immature neutrophils, such as myelocytes, metamyelocytes, and band cells into the circulation, known as ‘the left shift’ (115). Clinically, the left shift is defined by leucocytosis with the appearance of immature neutrophil precursor cells in the peripheral blood, which are normally only present in the BM. In sepsis, the presence of immature neutrophils in the circulating blood is often used as a clinical indicator (116, 117). In humans, mature and immature neutrophils can be distinguished based on the CD10 expression which is restricted to mature neutrophils (54). Furthermore, based on expression levels of CD16 and CD62L (L-selectin) neutrophils can be subdivided into mature, immature, and hyper-mature neutrophils. In non-inflammatory conditions, nearly all circulating neutrophils have a mature phenotype and are CD16^high^CD62L^high^ cells. In inflammatory settings, an increased number of immature neutrophils (CD16^dim^CD62L^high^) and hypersegmented neutrophils (CD16^high^CD62L^dim^) are found (118–120). Recently, a single-cell sequencing experiment was performed in a bacterial infection-induced mouse model. In this study, researchers showed that emergency granulopoiesis is associated with an augmented proliferation of the early-stage neutrophil progenitor cells in the BM and accelerated post-mitotic maturation of the neutrophils. Interestingly, the overall neutrophil differentiation in the BM remains intact but a substantial difference in the transition between the subpopulations was reported (57).

**Figure 8:**
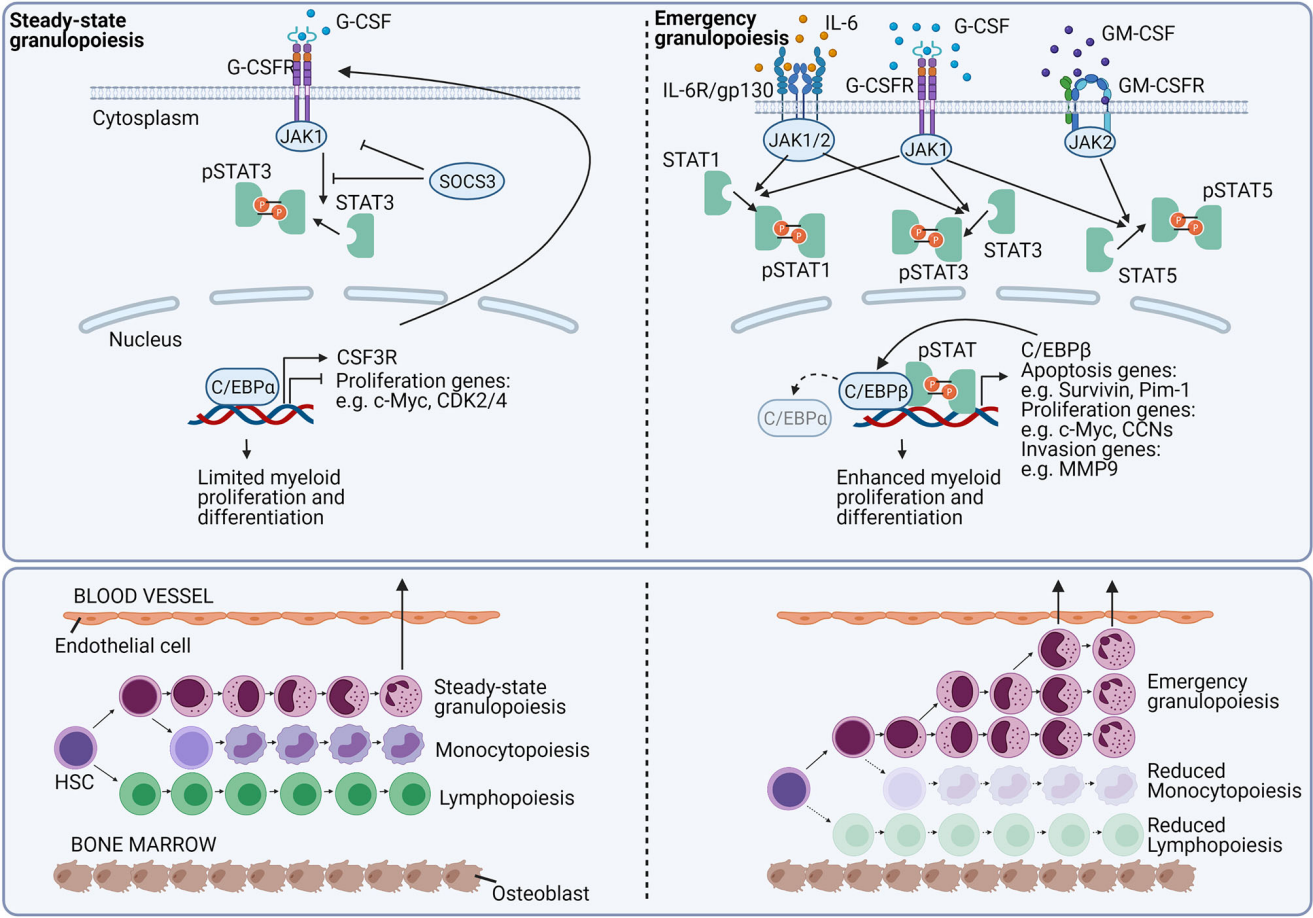
Growth factor signaling regulates emergency granulopoiesis. In homeostatic conditions, the granulocyte colony-stimulating factor (G-CSF) is the main growth factor that regulates neutrophil development. G-CSF signals *via* the G-CSF receptor (G-CSFR) (encoded by the CSF3R gene), and signals *via* the Janus kinase (JAK)-signal transducer and activator of transcription (STAT) pathway. *Via* unknown mechanisms, G-CSFR signaling induces the CCAAT-enhancer-binding protein-α (C/EBPα). C/EBPα is the major transcription factor involved in myelopoiesis. The transcription factor enhances the expression CSF3R, whereas it inhibits genes required for the cell cycle, eventually causing a limited myeloid proliferation and differentiation. During excessive inflammation, neutrophils massively migrate to the site of inflammation. To counterbalance neutrophil depletion and to provide newly needed neutrophils, emergency granulopoiesis is initiated. Emergency granulopoiesis is characterised by a large-scale *de novo* generation of new neutrophils from neutrophil progenitor cells. Interleukin-6 (IL-6), G-CSF, and granulocyte-macrophage colony-stimulating factor (GM-CSF) are mainly stimulating the proliferation and differentiation of new neutrophils. IL-6 binds the IL-6 receptor (IL-6R)/gp130 and signals in a JAK-STAT1 or STAT3-dependent way. Excessive G-CSFR signalling induces both STAT1, STAT3, and STAT5 phosphorylation and signalling, whereas GM-CSF receptor (GM-CSFR) signalling mainly tempts STAT5 activation. Upon activation, the pSTAT molecules are translocated to the nucleus where they directly stimulate genes involved in the regulation of apoptosis, proliferation, and cellular translocation. Additionally, pSTAT signalling stimulates the expression of the transcription factor C/EBPβ. C/EBPβ replaces C/EBPα, and releases the brake on proliferative genes, cranking myelopoiesis. During emergency granulopoiesis, not only mature neutrophils are leaving the BM, but also more immature neutrophils are being released. This process is called “left shift”.

Today, it remains challenging to accurately discriminate between steady-state and emergency haematopoiesis. Studies aiming to dismantle the mechanisms involved in emergency granulopoiesis have shown that granulopoiesis can be induced by cytokines in the absence of C/EBPα and suggested alternative pathways under emergency conditions (**Figure 8**) (121). It was found that especially C/EBPβ plays a crucial role in the stress-induced haematopoiesis, which was hampered in C/EBPβ KO mice (16, 121–127). Whereas both C/EBPα and C/EBPβ share downstream genes involved in the granulocyte differentiation (128), a less pronounced cell cycle inhibition was linked to C/EBPβ as compared to C/EBPα (37, 121, 129–131). The importance of C/EBPβ in stress-induced granulopoiesis has been confirmed in diverse mouse models and a zebrafish model (132–134). Interestingly, in chronic myeloid leukaemia, the breakpoint cluster region-Abelson murine leukaemia virus (BCR-ABL) fusion protein may drive a myeloid expansion by activating the emergency granulopoiesis in a C/EBPβ-dependent way (123, 124).

During extramedullary myelopoiesis neutrophils are formed outside the BM. The extramedullary myelopoiesis mainly takes place in spleen, and liver and, more controversially, also in lymph nodes, lungs, and kidneys (16, 135–139). Under acute or chronic stress conditions such as infection, these HSPC can seed in the extramedullary tissues (138). In the murine spleen, the HSPCs were localised close to transcription factor 21 (Tcf21)-positive stromal cells that can secrete niche factors such as CXCL12 and stem cell factor to support the splenic extramedullary haematopoiesis EMH (140). Although the BM niche and splenic stroma are quite different, neutrophil development in the spleen during extramedullary haematopoiesis is believed to follow the same hierarchical developmental order as described in the mouse BM. In this way, the spleen provides a unique reservoir able to supply additional myeloid cells during the immune challenge (49, 141, 142).

Emerging evidence from cancer studies suggests that neutrophils generated from the BM are functionally different from those derived from extramedullary sites such as the spleen. Driven by the cancer microenvironment, HSPCs can generate myeloid-derived suppressor cells (MDSC) in the spleen that can suppress the activation and proliferation of T cells (141, 143, 144). Interestingly, C/EBPβ can regulate the expression of enzymes such as arginase and inducible nitric oxide synthase both of which are required for the lymphocyte inhibitory activities of the MDSC (56) and the absence of C/EBPβ could reduce the tumour metastasis (145, 146). Pre-existing differences in the chromatin landscape between the different neutrophil maturational stages may contribute to observed heterogeneity since the same environmental trigger may induce a different biological output (111).

The term ‘reactive granulopoiesis’ has been proposed in settings where emergency granulopoiesis is induced by a non-infectious trigger such as described in active sJIA patients. sJIA patients show an increased number of circulating (immature) neutrophils which is in line with the peripheral expansion of immature CD34^+^CD33^+^ myelomonocytic precursors (147, 148). In addition, increased levels of neutrophil-derived mediators such as S100 proteins, MMP-8, MMP-9, elastase, and proteins involved in adhesion and chemotaxis of neutrophils (*e.g.* soluble E-selectin and soluble ICAM-1) were measured in the plasma of patients with sJIA. Furthermore, a positive correlation was found between increased numbers of neutrophils and levels of the inflammatory mediators C-reactive protein (CRP), ferritin, S100A8/A8, S100A12, MMP-8, and soluble E-selectin (149). Since neutrophils are thought to drive the pathogenesis of sJIA, in the next paragraph, we describe how the different neutrophil functions including the different neutrophil subsets may contribute to the disease pathogenesis of sJIA.

### How Neutrophilia May Fuel the Pathogenesis of sJIA

sJIA is a childhood autoinflammatory disease characterised by expansion of neutrophils. The disease can also occur in adults, where it is called Adult-onset Still’s disease (AOSD) (150). Apart from an important neutrophilia, patients are diagnosed by the presence of arthritis in one or more joints with or preceded by a quotidian fever of at least 2 weeks and accompanied by an erythematous rash, and enlargement of the lymph nodes (lymphadenopathy), liver and/or spleen (hepatosplenomegaly) or serositis (151). Patients also present with fatigue, abdominal pain, and weight loss (148, 152–154). In addition to neutrophilia, laboratory findings include (normo- or microcytic) anaemia, thrombocytosis, and a high erythrocyte sedimentation rate (ESR). Plasma levels of inflammatory cytokines (IL-6, IL-18), acute phase proteins S100A8/A9, and S100A12, and CRP are highly increased. In addition, the level of ferritin and D-dimers and liver enzymes such as aspartate transaminase (AST), alanine transaminase (ALT), and lactate dehydrogenase (LDH) are often elevated as well (148, 152, 154).

The exact aetiology of sJIA remains enigmatic. sJIA is not an infectious disease since, up till now, the condition has not been consistently associated with any pathogen. Given a seasonal variation in some studies, it has been postulated that infectious agents may trigger an excessive immune reaction to a relatively harmless trigger in genetically susceptible children (148, 152, 155). Clinically, sJIA follows a biphasic clinical course, in which the innate immune system is mainly involved at disease initiation (febrile stage) with excessive activation of neutrophils, monocytes, natural killer (NK) cells, and γδ T cells (156). The constitutive activation of the innate immune system supports the notion that describes sJIA as an autoinflammatory disorder. Several immunological genetic polymorphisms have been demonstrated to be associated with sJIA, defining sJIA as a multigenic and multifactorial autoinflammatory disease (157–169). In contrast to monogenic autoinflammatory diseases, also the adaptive immune system is involved in the pathogenesis of sJIA and contributes to the development of arthritis at a later stage (arthritic stage). Evidence for involvement of adaptive immunity in the pathogenesis of the disease is further provided by the fact that expression of the human leukocyte antigen (HLA) variant HLA-DRB1*11 places paediatric individuals at risk for developing sJIA.

Neutrophils from sJIA patients show a primed phenotype, characterised by an increased intracellular *ex vivo* ROS production upon formyl peptide stimulation and by an enhanced secretory vesicle degranulation (170). The increased degranulation was demonstrated by the increased surface expression of the complement receptor CD35, upregulation of the high-affinity Fcγ receptor CD64, and by the enhanced release of S100A8/A9 upon stimulation with phorbol 12-myristate 13-acetate (PMA) (149). Neutrophils from patients with AOSD also show upregulation of CD64 (170). It remains unknown if neutrophil-derived ectosomes are also altered in the sJIA pathogenesis. NETs may have a dual role in autoimmune and autoinflammatory diseases. As a disease tempering role, they catch pro-inflammatory cytokines and proteolytically degrade them to resolve the inflammation (171). In contrast, NETs are also highly immunogenic and were linked to the development of several autoimmune diseases including RA and systemic lupus erythematosus (SLE) (172–180). In sJIA patients, NETosis was never directly studied *ex vivo*. Nevertheless, increased serum histone levels in active sJIA patients compared to inactive patients or healthy controls (HCs) were described, arguing for enhanced NETosis. In addition, the serum histone levels were correlating to the sJIA disease activity (181). The increased levels of high mobility group box 1 protein (HMGB1) in patients with sJIA (182) is linked to enhanced NETosis since Garcia-Romo and colleagues have demonstrated in SLE that HMGB1 is released during NETosis, forming a positive feedback loop (183).

In sJIA, diverse clinical features in the febrile and the arthritic stage may be linked to neutrophils. At the initial stages, neutrophils are an important source of pro-inflammatory cytokines, chemokines, molecular mediators, proteases, and growth factors as blood neutrophil transcriptome analysis showed an inflammatory gene expression profile. Upregulated genes included neutrophil granule proteins, members of the IL-1 cytokine family, components of inflammasomes, the high-affinity IgG receptor CD64, CXCL8, and genes involved in the NF-κB pathway. Interestingly, the gene expression profile was partially overlapping with the transcriptome of sepsis (149, 184, 185). Additionally, using the sJIA mouse model we demonstrated that neutrophils are the main source of IL-1β (unpublished results from our own group). Worthy of note is the fact that neutrophils have a relatively high steady-state expression of pro-IL-1β and do not necessarily need a priming signal. Interestingly, patients displaying high numbers of neutrophils or neutrophil-associated genes appear to benefit from treatment with IL-1-targeting drugs such as anakinra and canakinumab (149, 168, 186). In addition, neutrophils provide an important source of alarmins or DAMPs, further amplifying the activation of the innate immune system. In sJIA, there is a growing interest in these molecules, in particular in the family of S100 proteins. S100 proteins may activate innate immune cells, predominantly monocytes and macrophages, upon binding the RAGE receptor or TLR4 by enhancing the secretion of pro-inflammatory molecules (187). High concentrations of S100 proteins (both S100A8/A9 and S100A12) are measured in patients with sJIA and therefore these molecules were proposed to be biomarkers (188–195). The release of IL-1 and other inflammatory cytokines by total white blood cells (WBCs) or monocytes from sJIA patients was reduced upon depleting serum S100A8/A9 or by preventing the S100A12 complex formation (191, 196, 197).

In the arthritic stage of sJIA, neutrophils may drive the development of arthritis fitting the remnant epitopes generate autoimmune (REGA) model that states that cells and molecules of the innate immune system (including neutrophils) can start the autoimmune reaction by cytokine-regulated proteolysis yielding remnant epitopes (2). Indeed, activated neutrophils can destroy the cartilage by *e.g.* releasing granule proteins or ROS production (198, 199). This was mainly investigated and reported in rheumatoid arthritis (RA) (172, 200, 201). Additionally, neutrophils express a functionally active membrane-bound RANKL, the ligand for receptor activator of NF-kB (RANK) and so can activate osteoclastogenesis (202). In the joints of JIA patients, activated neutrophils were abundantly present (203–205) and the presence of S100A12 in synovial fluids from JIA patients (189) provides evidence for their involvement in disease pathogenesis.

### Neutrophil Subsets in sJIA Patients

In sJIA patients, an increased number of immature CD16^dim^ neutrophils has been reported. The increased percentage of immature neutrophils in the peripheral blood cell count (banded neutrophils and granulocyte precursors) was confirmed microscopically (149). Also, patients with sJIA had a higher proportion of CD62L^low^ neutrophils compared to healthy controls (149, 184). The shedding of CD62L, mediated by membrane-proximal cleavage, is indicative of priming (as described above) (103, 206–208). Whereas one study failed to show an increased number of hypersegmented neutrophils, another study showed, by imaging cytometry, that patients with systemically active disease have increased numbers of hypersegmented neutrophils (149, 184). We recently uncovered neutrophil-DC hybrid cells (expressing both neutrophil and DC markers) in the synovial fluid from patients with JIA (205) that may serve as antigen-presenting cells (209, 210), eventually contributing to the arthritic phenotype. Remarkably, neutrophil protease genes (including *MMP-8*, and *MMP-9*) could also be found in PBMC microarray datasets of sJIA patients and might reflect the presence of low-density granulocytes in the PBMC fraction, which was confirmed by flow cytometry analysis (185). Remark that also in AOSD, increased levels of low-density granulocytes were reported, correlating with disease activity (211). Single-cell sequencing on splenic neutrophils derived from the sJIA-like mouse model recently showed the emergence of MDSCs. From this, it is tempting to speculate that neutrophils, next to their pro-inflammatory properties, also may have some disease-tempering effects in sJIA (Malengier-Devlies et al., unpublished results).

## Cytokines That Link Neutrophils to sJIA Pathogenesis

Diverse cytokines and growth factors play an important role in the pathogenesis of sJIA and all of them regulate important aspects of neutrophil homeostasis (16, 212–216). In a next part of this review, we overview the different neutrophil-regulating cytokines and growth factors in sJIA (including G-CSF, GM-CSF, IL-17, IL-1β, IL-6, IL-18, and IFN-γ). Considering the current beneficial effects of agents blocking IL-1- and IL-6 in sJIA (217–228), a better understanding of all neutrophil-regulating cytokines and growth factors may open new avenues for therapeutic intervention.

### G-CSF

G-CSF is the main haematopoietic growth factor required for the proliferation and differentiation of haematopoietic precursor cells into neutrophils (229–234). G-CSF regulates the commitment of progenitor cells to the myeloid lineage (235), induces the proliferation of granulocytic precursor cells (236), reduces the transit time through the granulocytic compartment (236), and controls the viability of the BM neutrophil pool (233, 234, 237). Furthermore, it induces the release of mature neutrophils from the BM into the blood by the internalisation and consequent downregulation of CXCR4 (238, 239) or by downregulation of CXCL12 in the BM (**Figure 4**) (240). In contrast, G-CSF can also impede the CXCR2-induced neutrophil mobilisation by negatively regulating CXCR2-mediated intracellular signalling which under specific bacterial infections, functions as a negative regulator of neutrophil mobilisation (241). Both *in vitro* and *in vivo*, G-CSF acts on mature neutrophils and may enhance ROS production, adherence, phagocytosis, killing, antibody-dependent cellular cytotoxicity (ADCC), and may induce phenotypic alterations such as increased expression of CD11b (242). G-CSF also affects the expression of the pro-survival protein survivin which increases the lifespan of mature neutrophils (243, 244).

G-CSF signals *via* the homodimeric G-CSF receptor (G-CSFR) (245) in a manner that depends on a Janus kinase (JAK)/signal transducer and activator of transcription (STAT). G-CSF can stimulate three members of the STAT family (STAT 1, 3, and 5) (246–250). However, the role of STAT1 and STAT5 in the granulopoiesis is limited (251–256) and myeloid differentiation is mainly induced by STAT3 (257). In steady-state conditions, the G-CSFR signalling is controlled in a suppressor of cytokine signalling 3 (SOCS3)-dependent way (258) and STAT3-deficient mice are marked by peripheral neutrophilia (259–262). In steady-state granulopoiesis, the expression of G-CSFR is regulated by C/EBPα. C/EBPα drives the expression of many genes that encode proteins required for myeloid progenitor proliferation and granulocyte differentiation. Besides, it restricts excessive proliferation of neutrophil precursors by inhibiting the expression of genes required for cell cycle progressions *e.g.* the genes encoding MYC, cyclin-dependent kinase 2 (CDK2), or CDK4 (16, 263). During emergency granulopoiesis, when G-CSF levels are markedly increased, STAT3 is directly stimulating the expression of MYC and C/EBPβ, which in turn further stimulates MYC transcription by replacing C/EBPα at the promoter region (**Figure 8**) (16, 133).

G-CSFR-deficient mice have lower amounts of immature granulocytic precursors and neutrophils in the BM, show a 70-80% reduction of circulating neutrophils (due to a defective release), and are defective in certain mature neutrophil cell functions (233, 234, 264). Similarly, humans expressing dominant-negative receptor mutations in *G-CSFR* are neutropenic (265, 266). Additionally, mutations in the haematopoietic cell-specific Lyn substrate 1-associated protein X1 (HAX1), which contributes to the G-CSFR signalling pathway cause severe neutropenia in humans (267, 268). Interestingly, emergency granulopoiesis, however, can occur in a G-CSF-independent way and G-CSFR-deficient mice can still mount a granulopoietic response in a sterile model of peritonitis or during a *C. albicans* infection. In contrast, G-CSFR KO mice showed a more severe disease during infection with *L. monocytogenes* (234, 269–272).

The exogenous administration of G-CSF mimics the physiological responses that are observed during emergency granulopoiesis and increases the neutrophil count in peripheral blood (114, 273). Upon G-CSF treatment, also HSCs can be mobilised from the BM into the blood (274, 275). Currently, G-CSF is used in clinics to treat neutropenic individuals (276–278) or to mobilise hematopoietic progenitors for transplantation (230, 279).

Many tissues including endothelial cells, macrophages, epithelial cells, fibroblasts, and BM stromal cells produce G-CSF when stimulated with inflammatory stimuli such as LPS, IL-1β, or TNF-α (280–283). IL-17 and IL-23 have been implicated as major upstream regulators of G-CSF (discussed below) and play a crucial role in the pathogenesis of sJIA (230, 281, 284). G-CSF levels are increased in sJIA patients compared to HCs, whereas in other JIA subtypes, G-CSF was demonstrated to be a major regulator of the neutrophil gene expression signature (168, 185). In line with the pathological role of neutrophils in sJIA, the highest levels of G-CSF were found in patients with an incomplete response or nonresponse to anakinra (168).

In addition to its neutrophil regulatory properties, G-CSF induces the release of prostaglandin E2 and induces fever, one of the hallmark clinical features in sJIA (285). Furthermore, G-CSF can stimulate the peripheral sympathetic nervous system to release catecholamines (84) which may reduce the number of osteoblasts and the production of CXCL12 (286, 287). Interestingly, G-CSF is a strong inhibitor of the NK cell function, altering the receptor expression profiles and reducing the cytotoxic and cytokine producing capacity of these cells (288, 289). It is important to note that NK cells are an important group of innate immune cells involved in the pathogenesis of sJIA. The role of NK cells in sJIA has recently been reviewed by Vandenhaute et al. (290). The mutual role of both NK cells and G-CSF in sJIA requires further investigation. Using a sJIA-like mouse model, we recently showed that G-CSF regulates the development of arthritis. Additionally, we demonstrated that G-CSF stimulates extramedullary myelopoiesis in the spleen. Neutrophils followed a similar differentiation and maturation path as described in the BM, which was found to be CEBP/β-driven (Malengier-Devlies et al., manuscript under revision).

### GM-CSF

Granulocyte-macrophage colony-stimulating factor (GM-CSF) is a cytokine secreted by a variety of cells. The major sources of GM-CSF include activated T and B cells, monocytes/macrophages, endothelial cells, fibroblasts but also neutrophils, eosinophils, epithelial cells, mesothelial cells, chondrocytes, Paneth cells, and tumour cells may secrete GM-CSF (291–293). In T cells, the release of GM-CSF is triggered by IL-1β and IL-23 or IL-1β and IL-12 in mice and humans respectively (294–296). In fibroblasts, endothelial cells, chondrocytes, and smooth muscle cells, TNF-α and IL-1 are the main inducers of GM-CSF production whereas in macrophages/monocytes GM-CSF is predominantly released upon TLR-stimulation (291). GM-CSF can form a positive feedback loop by activating macrophages and DCs to produce IL-23, IL-1β, and IL-6 activating Th17 and Th1 cells that in turn express GM-CSF (297). The production can be inhibited by IFN-γ (298), IL-4 (299), IL-10 (300), and glucocorticoids (301).

GM-CSF is required for the *in vivo* development of neutrophils, monocytes, and macrophages from BM precursor cells and plays a crucial role in the maintenance of the innate immune homeostasis (302–305). Depending on the dose, GM-CSF can have different effects on myeloid cell survival, proliferation, or differentiation (243, 306–308). GM-CSF also upregulates the antimicrobial function of mature neutrophils and enhances ROS production, adherence, killing, phagocytosis, and antibody-dependent cellular cytotoxicity both *in vitro* and *in vivo* (242, 309). Furthermore, GM-CSF phenotypically alters neutrophils and can upregulate the expression of the integrin CD11b, which facilitates adhesion and tissue entry (242, 310). Following chemo- or radiotherapy, exogenous GM-CSF can be used to restore the myeloid populations (311). In addition to its effect on neutrophils, GM-CSF stimulates the activities of macrophages, DCs, and B cells [reviewed in (312)].

GM-CSF signals *via* the GM-CSF receptor (GM-CSFR) that is composed of a low-affinity α chain and a high-affinity β chain. The β chain is shared with IL-3 and IL-5 receptors (307). GM-CSFR is expressed on myeloid cells and some non-hematopoietic cells, but not on T cells (313). Four main signalling pathways can be triggered by G-CSFR (314–316). The main pathway involves JAK2/STAT5 signalling and facilitates the activation of genes such as pim-1, CIS, and cyclin to induce cell myeloid differentiation and proliferation (317–319). Next, both the phosphatidylinositol-3-kinase (PI3K) and JAK/STAT-Bcl-2 signalling pathway are involved in cell survival (320). Eventually, the ERK1/2 and NK-κB pathways mediate cell differentiation and inflammation (321, 322).

Whereas GM-CSF-deficient mice have an impaired reproductive capacity and develop pulmonary alveolar proteinosis (PAP), GM-CSF-deficient mice have a normal basal granulopoiesis and show no alterations in peripheral blood counts (271, 323, 324). This indicates that other growth factors aside from GM-CSF have a redundant role in myeloid cell development and differentiation under steady-state conditions. In emergency granulopoiesis, GM-CSF plays an important role and GM-CSF-deficient mice fail to control infections with *L. monocytogenes* or *M. avium* (270, 325). In contrast, mice lacking the three myeloid colony-stimulating factors [G-CSF, GM-CSF, and macrophage colony-stimulating factor (M-CSF)] still mount an inflammatory response in a sterile model of peritonitis (272). Interestingly, GM-CSF (but also IL-3 and IL-6) can restore the lack of G-CSFR expression in C/EBPα-deficient mice and can initiate a C/EBPα-independent granulopoiesis (326–328). *In vitro* studies suggested C/EBPβ as the driving transcription factor in this process, since C/EBPβ-deficient hematopoietic cells had impaired responsiveness to GM-CSF (132).

GM-CSF has a pathological role in Th17-driven autoimmune diseases such as multiple sclerosis (MS) and RA (294, 297, 329). Consequently, the ablation of GM-CSF signalling could suppress the disease in models of arthritis, multiple sclerosis (MS), and lung disease (308, 330–332). In patients with JIA, the frequency of GM-CSF producing T helper cells was significantly increased in the synovial fluid and correlated with an increased ESR (333, 334). sJIA patients show increased plasma levels of GM-CSF as compared to healthy controls and a significantly decreased level was observed in the anakinra responder group (80). In the sJIA-like mouse model, depleting GM-CSF however had no effect on the observed disease symptoms (unpublished results from our laboratory).

### IL-17

IL-17 is a pro-inflammatory cytokine that is mainly secreted by Th17 cells. Th17 cells are induced from naïve T cells in the presence of both TGF-β and IL-6 (in mice) or IL-1β (in humans). The pro-inflammatory cytokines TNF-α and IL-1β can synergistically increase IL-6 production, further contributing to Th17 cell differentiation. Following activation, RORγt is induced, which promotes the expression of IL-17 and the IL-23 receptor (IL-23R). Subsequent IL-23-signalling further increases the RORγt and IL-17 expression in a STAT3-dependent way (335). Although IL-17 was first described as a T cell-secreted cytokine, it can also be produced by innate immune cells such as γδ T cells (336). Since these cells do not require induction of the IL-23R, these cells may induce a fast IL-17 response to IL-1β or IL-23 without any T cell receptor engagement (337). Note that together with IL-1β, S100 proteins may promote Th17, γδ T cell development, or induce IL-17 expression in autoreactive CD8^+^ T cells, sustaining a potentially important amplification loop mediated by activated neutrophils (197, 338–340).

In sJIA patients, plasma or serum levels of IL-17 were found to be either normal or increased compared to those of HCs (168, 340, 341). Intracellular flow cytometric staining showed that IL-17 was increasingly expressed in circulating γδ T cells of patients with sJIA. Furthermore, an increased number of IL-17-producing T cells in patients was reported (340, 342). Using the sJIA-like mouse model, we showed that IL-17 is a major cytokine driving the disease pathogenesis (343). In analogy, in patients with sJIA, the development of anaemia was linked to IL-17 since a positive correlation was seen between circulating IL-17 and the erythropoiesis signature (344). In addition to the development of anaemia, IL-17 expression may explain multiple disease symptoms including arthritis, fever (*e.g.* by the release of prostaglandins such as PGE2), and neutrophilia (148, 345–348).

Already since the first reports describing IL-17, an indirect role of the cytokine on neutrophil proliferation and differentiation was demonstrated (281). IL-17 mainly functions on epithelial, endothelial, and stromal cells, inducing the expression of pro-inflammatory cytokines, growth factors, and chemokines that regulate granulopoiesis, recruitment, and life span of new neutrophils (349). IL-6 was the first identified target gene downstream of IL-17, regulating granulopoiesis (350). Furthermore, IL-17 induces the production and release of the two main granulocytic growth factors, namely G-CSF and GM-CSF (281, 351, 352). Since IL-23 is the major regulator of IL-17 release and subsequent G-CSF induction, its pathway is often referred to as the “IL-23-IL-17-G-CSF axis” (353). Remark that this axis also plays a central role during neutrophil homeostasis. Here, phagocytosis of dying neutrophils by specialised macrophages in the tissue *i.e.* lung, BM, or spleen, may block the secretion of IL-23 and subsequent IL-17-regulated neutrophil release (described above) (112, 354). IL-17 also induces the production of other pro-inflammatory cytokines including TNFα, IL-1β as well as cyclooxygenase 2 (COX2), and inducible nitric oxide synthase (iNOS), which all directly or indirectly regulate the formation of new neutrophils (355). Next, IL-17 also regulates the attraction of neutrophils *via* the induction of various chemokines *i.e.* CXCL1 (KC), CXCL2 (MIP2), CXCL6 (GCP-2), or CXCL8 (IL-8) (356). Furthermore, IL-17 can stimulate endothelial expression of P-selectins, E-selectins, and integrin ligands including ICAM-1 and VCAM, enhancing the neutrophil mobilisation (357). Remark that neutrophils themselves can form a positive feedback loop during neutrophil recruitment by releasing pro-inflammatory cytokines and chemokines (8). Unfortunately, no reports exist on the use of IL-17 blocking agents (such as secukinumab, ixekizumab, or bimekizumab) in treating sJIA patients nor on its role in driving neutrophilia.

### IL-1β

IL-1β is a cytokine with many pro-inflammatory activities. Several inflammatory cell types including activated monocytes, neutrophils, or macrophages secrete the cytokine *via* a two-step mechanism. PAMPs or damage-associated molecular patterns (DAMPs) induce the transcription of pro-IL-1β. Subsequent processing into active IL-1β by inflammasomes requires the presence of a second stimulus (358). IL-1β can bind its IL-1R type I (IL-1RI) that, together with the IL-1 receptor accessory protein (IL-1RAcP), induces MyD88-dependent signalling (359).

The pro-inflammatory activity of IL-1β is further regulated by the naturally occurring IL-1 receptor antagonist (IL-1Ra) and by IL-1 receptor type 2 (IL-1R2) (360). IL-1Ra blocks the binding of IL-1 to its signalling receptor. IL-1R2, which is mainly expressed on neutrophils and their precursors, lacks the signalling Toll/IL-1R domain and regulates the pro-inflammatory activity of IL-1β by acting as a decoy receptor (361, 362). The IL-1R2 has a higher affinity for IL-1 than for the IL-1Ra and thus further enhances the anti-inflammatory function of IL-1Ra (363). The surface expression of IL-1R2 is tightly regulated. Anti-inflammatory molecules such as glucocorticoids may augment the surface expression of IL-1R2 and may in part explain the beneficial effects of glucocorticoid treatment in sJIA (364, 365). Pro-inflammatory molecules such as ROS, LPS, TNF-α, leukotriene B4, and fMLF can initiate a rapid proteolytic cleavage of the membrane-bound IL-1R2 by different proteases (364, 366).

Myelopoiesis is stimulated both directly and indirectly upon binding of IL-1β to its receptor. IL-1β itself or in synergy with other growth factors (*e.g.* G-CSF), can induce the proliferation and differentiation of HSPCs and GMPs. This induction is based on the activation of PU.1 and can be blocked in IL-1R1-deficient mice (367, 368). In naïve conditions, IL-1R1-deficient mice have no defects in myeloid cell numbers (369). Alternatively, IL-1β can indirectly regulate granulopoiesis by modulating the production of neutrophilic growth factors and inflammatory mediators including IL-3, IL-6, G-CSF, and GM-CSF (121, 370–372). More importantly, IL-1β - together with IL-23 - is a potent inducer of IL-17 expression in CD4^+^ T cells or γδ T cells (337, 373). IL-1β additionally regulates the recruitment of neutrophils by the production of neutrophil attracting chemokines such as CXCL1, CXCL2, or CXCL8 and by the induction of adhesion molecules on endothelial cells (370, 374, 375). Interestingly, IL-1β can also directly prime neutrophils for ROS production or NET formation and may prolong their lifespan (149, 244, 376–378).

Many of the symptoms observed in sJIA patients including fever, rash, thrombocytosis, neutrophilia, and arthritis, can be explained by the increased production of IL-1β (359). However, plasma IL-1β levels are hard to measure and multiple studies failed to show increased plasma or serum IL-1β levels (340, 341, 379–382). Neither was an increased *IL-1β* gene expression profile observed in patients (383–385). Increased IL-1β production was demonstrated upon stimulation of PBMCs with serum of sJIA patients. However, a reduced IL-1β secretion by monocytes of sJIA patients was reported, suggesting that other cellular sources, such as neutrophils, might play an important role in the production of IL-1β in these patients (168, 218, 386). The successful treatment with IL-1-blocking agents such as anakinra, canakinumab, and rilonacept was the first proof of the importance of IL-1 in sJIA (218, 222, 381, 387, 388). IL-1 blocking therapies are reported to be equally beneficial as first-line therapy, underlining the importance of IL-1β at the disease onset (221, 389). This is in line with the “window of opportunity” that has been proposed by Nigrovic (345). A localised action of IL-1β or activity at low levels might explain the absence of an elevated plasma IL-1β signature (148). Follow-up studies and the identification of single nucleotide polymorphisms (SNPs) in IL-1-related genes have also pointed towards a pathological role for IL-1β in sJIA (158, 168, 218, 384).

In sJIA, the number of neutrophils was rapidly normalised during disease remission and after treatment with anakinra (149). A high (immature) neutrophil count was found to correlate with a good response to anakinra and a short disease duration assuming that the effect of anakinra is mainly due to its effects on neutrophils (149, 168). Indeed, IL-1β has been demonstrated to be an important cytokine in the priming of neutrophils, which could be reverted upon anakinra treatment (149). Also in patients with AOSD, a strongly elevated neutrophil number was associated with an IL-1 gene expression profile and a pronounced upregulation of canakinumab-responsive genes (390).

### IL-6

IL-6 can be produced by almost all stromal and immune cells in response to *e.g.* IL-1β, TNF-α, or TLR ligands. IL-6 binds the IL-6 receptor (IL-6R) which is expressed on a wide variety of cell types and signals in a JAK-dependent way (391, 392).

Although IL-6 is not necessary for maintaining neutrophil homeostasis (393), the cytokine plays a critical role during emergencies (394–396) and may stimulate granulopoiesis in the absence of both G-CSF and GM-CSF (371). Administration of IL-6 induces a biphasic neutrophilia in animals *via* a rapid mobilization of neutrophils from the marginated pool into the circulation (397–399), followed by an accelerated release of neutrophils from the bone marrow, which is induced by a stimulated myeloid cell differentiation (371, 397, 400, 401). IL-6 deficient mice show an impaired neutrophil response after *C. albicans* infection (402) and patients treated with the humanised monoclonal anti-IL-6R antibody tocilizumab, show transient neutropenia, all demonstrating the neutrophil mobilising properties of IL-6 (403–408). Interestingly, in C/EBPα KO mice, IL-6 can induce neutrophil differentiation by restoring the G-CSF receptor expression (16, 327).

Classically, IL-6 signals upon binding to its membrane-bound IL-6-receptor alpha subunit (IL-6Rα), resulting in gp130 homodimerization, phosphorylation of STAT3 and STAT1 proteins, and downstream signalling (409). IL-6 can also promote IL-6 *trans-*signalling in cells that express gp130 but lack the IL-6Rα which requires the binding of IL-6 to the soluble IL-6Rα (sIL-6Rα) into an IL-6/IL-6Rα complex. Neutrophils themselves are an important source of sIL-6Rα. IL-6Rα can proteolytically be cleaved *via* the TNFα converting enzyme-like enzyme (410) upon stimulation with CRP, CXCL8, C5a, LTB_4_, or platelet-activating factor (PAF) (410). In this way, neutrophils promote IL-6 *trans*-signalling in other cell types to favour the resolution of inflammation (411–413). Together with IL-6, the IL-6/IL-R complex activates endothelial cells to secrete monocyte chemoattractant protein-1 (MCP-1) (or CCL2) and induces the expression of adhesion molecules (such as ICAM1 or VCAM) to limit neutrophil accumulation while favouring monocyte recruitment (414, 415). This could explain why in animal models of inflammation, the neutrophilic infiltrate is more dominant in IL-6 KO than in WT animals (412, 416). The recruitment of monocytes is protective in acute models, whereas, during chronic inflammation such as CIA, colitis, or experimental autoimmune encephalomyelitis (EAE), IL-6 predominantly fulfils pro-inflammatory functions by favouring mononuclear-cell accumulation, angioproliferation, B cell maturation, and by promoting the anti-apoptotic properties of T cells (416–420).

Conflicting results regarding the direct role of IL-6 on neutrophil function have been reported (421–426). Recently, it was demonstrated that granulocytes are unable to induce STAT-signalling upon stimulation with IL-6 since the expression of gp130 is lost during the maturation of granulocytes (427). One may therefore speculate that the function attributed to IL-6R in neutrophils rather results from its effects on contaminating cell populations (428).

In sJIA, the gene expression of *IL-6* (383, 384) and IL-6 protein levels in serum were strongly elevated in patients compared to HCs (168, 340, 341, 379–381, 429–436). Furthermore, increased IL-6 levels were measured in the synovial fluid of sJIA patients (341). Interestingly, high IL-6 levels were associated with more active joint inflammation (437). Several sJIA symptoms - including fever, thrombocytosis, and growth impairment - can at least partially be explained by the elevated levels of IL-6. IL-6 may be responsible for the microcytic anaemia, by blocking the iron supply to the developing erythroid cells and induces the production of acute-phase proteins (*e.g.* CRP) in hepatocytes (148, 438–440). The crucial role of IL-6 in sJIA was confirmed by the successful use of the humanised anti-IL-6 receptor antibody tocilizumab (222, 223, 227). Tocilizumab treatment could induce neutropenia and increases the risk of infection in patients. However, these adverse effects were outweighed by the beneficial effect on the sJIA-like features (227). Interestingly, the treatment of sJIA patients with tocilizumab was associated with a significantly different neutrophilic gene expression profile and marked upregulation of genes associated with oxidative phosphorylation (441).

### IL-18

IL-18 is a pro-inflammatory cytokine constitutively expressed by monocytes, keratinocytes, and epithelial cells. Like IL-1β, the cytokine is produced in a premature form (pro-IL-18) that requires inflammasome-dependent cleavage by caspase-1 in order to become biologically active (442, 443). IL-18 binds the IL-18 receptor expressed on lymphocytes, DCs, and mesenchymal cells. The cytokine signals like IL-1β in a MyD88-dependent way and exerts its pro-inflammatory action increasing the levels of cell adhesion molecules, including chemokine production, and promoting joint inflammation (443). IL-18 is a potent inducer of IFN-γ and is often referred to as “IFN-γ-inducing factor”. IFN-γ, in turn, regulates the activity of IL-18 *via* the induction of an IL-18 endogenous inhibitor, the IL-18 binding protein (IL-18BP), thus setting up an auto-inhibition loop (442).

IL-18 has pleiotropic effects on neutrophil activation, including pro-inflammatory cytokine expression, degranulation, and priming of the oxidative burst (1, 444, 445). Since the activation of neutrophils induces inflammasome-mediated IL-18 release, the cytokine can trigger a positive activation loop (446). IL-18 has no direct effect on granulopoiesis, but can stimulate the secretion of CXCL1 and CXCL2, two chemokines involved in the recruitment of neutrophils (447). Indirectly, IL-18 may alter neutrophil homeostasis by inducing IFN-γ in T cells and NK cells (442).

High plasma levels of IL-18 were measured in active sJIA patients and have been proposed as a candidate biomarker (341, 379, 429, 437, 448). In contrast, the levels of IL-18BP were found only moderately increased, insufficient to counteract the high levels of IL-18 (449, 450). Also in patients with inactive disease, moderately increased levels of IL-18 were measured (341, 379, 429, 437, 448). IL-18 is considered as an important cytokine involved in the pathogenesis of sJIA (437). A Phase II clinical trial in which AOSD patients were treated with the IL-18-blocking recombinant IL-18PB (Tadeking alfa) showed a favourable safety profile and demonstrated clinical and laboratory efficacy in 50% of the treated patients. Interestingly, both the number of neutrophils as well as the neutrophil-associated S100A8/A9 and S100A12 protein plasma levels were significantly decreased upon treatment (451).

### IFN-γ

IFN-γ is a cytokine that is produced predominantly by activated T cells and NK cells upon stimulation with IL-12 and IL-18 (452–454). The cytokine binds to its specific IFN-γ receptor that signals in a JAK/STAT-dependent way. IFN-γ is a cytokine with both pro- and anti-inflammatory properties.

With respect to myelopoiesis, IFN-γ favours the production of monocytes at the expense of neutrophils (455, 456). Consistently, IFN-γ induces the differentiation of human progenitor cells into monocyts while blocking G-CSF-induced granulopoiesis (457). IFN-γ also inhibits neutrophil recruitment in an indirect way, by blocking the development of Th17 cells and by counteracting IL-17- induced neutrophil-related chemokines (458, 459). These findings explain why IFN-γ-deficient mice are marked by massive granulopoiesis during infection with *M. tuberculosis* or *T. gondii* (460, 461). Similarly, the sJIA-like mouse model requires an IFN-γ-deficient background and is hallmarked by a massive neutrophilia (343).

IFN-γ can also directly stimulate neutrophils e.g. by alterering the expression of genes involved in migration, chemotaxis, phagocytosis, or apoptosis (462). IFN-γ-stimulated neutrophils have a prolonged life span, an increased capacity for phagocytosis, oxidative burst, and NET formation, and show an enhanced pro-inflammatory cytokine expression (463, 464). In contrast, IFN-γ inhibits the expression of neutrophil-specific chemokines (*i.e.* CXCL8/IL-8) and the release of key neutrophil-derived soluble mediators (*i.e.* MMPs and serine proteases), to counteract inflammation-induced tissue damage (454, 465–467). IFN-γ also induces the expression of PD-L1 on neutrophils, which is involved in the suppression of lymphocyte proliferation (468). Besides, IFN-γ tempts the expression of genes involved in antigen-presentation (209, 462, 463).

In sJIA patients, the role of IFN-γ is incompletely understood. The levels of IFN-γ are moderately increased and are low in comparison to its upstream inducer IL-18 (168, 341, 379). An increased number of IFN-γ producing T cells was found in patients. However, a lower IFN-γ expression was measured in these cells. *In vitro* stimulation of PBMCs resulted in a decreased IFN-γ production in patients when compared to healthy individuals (340, 342). Likewise, sJIA patients display an absent IFN-γ gene signature in their PBMCs (147, 383, 384), NK cells (379), and synovial tissues (469). NK cells of sJIA patients produced less IFN-γ due to a defective phosphorylation of the IL-18 receptor upon signalling (379, 450). Since monocytes of sJIA patients were able to respond to IFN-γ, a limited *in vivo* exposure was hypothesized in patients (469). Considering the regulatory effect of IFN-γ on IL-17 activity and downstream G-CSF production, enhanced neutrophilia may be linked to this low IFN-γ exposure. Indeed, using our mouse model, we recently described a G-CSF-driven extramedullary myelopoiesis in the IFN-γ KO mice upon CFA-immunisation (Malengier-Devlies et al., manuscript under revision).

## Concluding Remarks

In homeostatic conditions, neutrophil accumulation and activation are tightly regulated *via* distinct regulatory mechanisms that include neutrophil granulopoiesis, release, storage, extravasation, and clearance. To meet the high demand for new neutrophils during severe (systemic) inflammation, emergency granulopoiesis can be induced by diverse yet partially redundant growth factors and cytokines. Given their antimicrobial properties, capacity to release soluble mediators, and direct cell interactions, this *de novo* generation of neutrophils might be lifesaving during infectious inflammation. However, in the context of autoimmunity, autoinflammation (including sJIA) or disproportionate infection-induced inflammation (including COVID-19) (470), excessive neutrophil production and activation might be destructive to the host. Results from scRNAseq and CyTOF have greatly improved our understanding of neutrophil ontogenesis in homeostatic conditions. However, additional research is needed to understand neutrophil development in emergency situations and at extramedullary sites. It remains to be established whether extramedullary-derived neutrophils have another functionality or plasticity than neutrophils generated in the BM. It will also be important to understand the functional differences between mature and immature neutrophils that emerge during the emergency granulopoiesis. Next, advanced insights on neutrophil functionality, plasticity, and overlap between diverse pathophysiological situations are warranted. For example, in sJIA, it remains particularly interesting to understand and compare the function and phenotype of neutrophils in the joints and blood. This may help us to understand their role in the pathophysiology and the discrimination between the arthritic and febrile phases of the disease. A better understanding of the different cytokines and growth factors regulating neutrophil homeostasis would enable the development of new targeted therapies that prevent uncontrolled tissue inflammation without impairing the anti-microbial function of neutrophils. Yet we are not able to discriminate between a direct effect of the cytokines and growth factors on the observed disease symptoms or an indirect role *via* its neutrophil-regulatory properties. Neutrophil depletion studies in the sJIA-like mouse model are an excellent tool to understand the role of neutrophils in the pathophysiology of sJIA. Unfortunately, depletions were incomplete and were followed by a fast rebound of new immature neutrophils (471). In sJIA, we might speculate whether, similar to what has been reported for IL-1β, there would exist a window of opportunity in which G-CSF-, GM-CSF-, IL-17- or IL-18-targeting drugs could be considered (*e.g.* in patients that do not respond to IL-1 or IL-6 therapies or during the arthritic stage of the disease). Next, we could also envisage studying the clinical effects of drugs targeting neutrophil migration. This could be drugs that block the main chemokine receptors expressed by neutrophils (*e.g.* CXCR1 and CXCR2), adhesion molecules (*e.g.* CD11b), or chemoattractants [*e.g.* IL-8 (CXCL8) in humans or GCP-2 (CXCL6), KC (CXCL1), and MIP-2 (CXCL2) in mice]. In conclusion, sJIA is a unique childhood autoinflammatory immune disorder characterised by massive neutrophilia. Neutrophils in sJIA have a hyperactivated phenotype and are thought to play an important role in the pathophysiology of sJIA. The exact role of neutrophils on sJIA-like features including its regulatory cytokines and growth factors are gradually being revealed but certainly require further investigation.

## Author Contributions

BM-D wrote the first draft of the manuscript and designed figures with Biorender software. MM, PP, CW, and PM critically read and edited the manuscript. All authors contributed to the article and approved the submitted version.

## Funding

This study received funding from the KU Leuven (C1 grant no. C16/17/010) and from FWO-Flanders (GOA3218N and GOC3420N). MM obtained a PhD fellowship supported by the L’Oréal – UNESCO for Women in Science initiative and the FWO-Vlaanderen. The funder was not involved in the study design, collection, analysis, interpretation of data, the writing of this article or the decision to submit it for publication.

## Conflict of Interest

CW obtained unrestricted grants to KU Leuven from Novartis, Roche, GSK immuno-inflammation and Pfizer.

The remaining authors declare that the research was conducted in the absence of any commercial or financial relationships that could be construed as a potential conflict of interest.

## Publisher’s Note

All claims expressed in this article are solely those of the authors and do not necessarily represent those of their affiliated organizations, or those of the publisher, the editors and the reviewers. Any product that may be evaluated in this article, or claim that may be made by its manufacturer, is not guaranteed or endorsed by the publisher.
